# Role of Growth Stage and Environmental Conditions in Root Rot Development and Grain Yield of Spring Wheat in the Almaty Region, Southeast Kazakhstan

**DOI:** 10.1155/tswj/6567136

**Published:** 2026-04-03

**Authors:** Vladimir Tsygankov, Alma Kokhmetova, Yerlan Dutbayev, Kanat Bakhytuly, Aidana Kharipzhanova, Madina Kumarbayeva, Zhenis Keishilov, Baktigul Raimbekova, Assiya Kokhmetova, Kanat Mukhametzhanov, Ardak Bolatbekova

**Affiliations:** ^1^ Kazakh Research Institute of Horse Breeding and Forage Production, Aktobe, Kazakhstan; ^2^ Institute of Plant Biology and Biotechnology, Almaty, Kazakhstan; ^3^ Kazakh National Agrarian Research University, Almaty, Kazakhstan; ^4^ Department of Biology, Natural Science Institute, Kazakh National Women′s Teacher Training University, Almaty, Kazakhstan

**Keywords:** agronomic backgrounds, breeding, fungicides, growth stages, harvesting, root rot, spring soft wheat, tillering, yield

## Abstract

In 2024, a study was conducted in the Almaty Region on 16 varieties of spring soft wheat to assess the impact of growth stages, cultivation conditions, and the severity of root rot infection on biometric parameters and crop yield. This study represents the first systematic evaluation of root rot dynamics across multiple growth stages and contrasting agronomic backgrounds in Kazakhstan. It was found that the maximum root rot prevalence (32.65%) and severity (8.78%) were observed during the tillering phase. By the harvesting phase, these indicators decreased, indicating the dynamic nature of pathogenesis. Cultivation conditions had a significant influence: Under natural conditions, disease prevalence was 26.79%, while on an infectious background, it increased to 31.60%. With fungicide application, prevalence decreased to 8.80%. Fungicide treatment proved effective in suppressing infection, though some varieties exhibited stress responses, leading to reduced productivity. The highest grain weight per plant (13.71 g) was recorded under natural conditions, while the lowest (8.31 g) was observed on the infectious background. Analysis of biometric parameters revealed significant differences between varieties in traits such as tillering capacity, stem length, spike length, and spikelet number. These findings provide a practical framework for improving integrated disease management strategies and for breeding wheat varieties adapted to regional agroecological conditions. These results highlight the importance of integrated disease management and genotype selection to sustain wheat productivity under biotic stress.

## 1. Introduction

Spring soft wheat (*Triticum aestivum* L.) is one of the most important grain crops that ensures food security and stability of the agroindustrial complex [1]. However, its productivity is significantly limited by the impact of biotic stressors, among which a special place is occupied by root rot caused by a complex of phytopathogens, including *Fusarium* spp., *Bipolaris sorokiniana*, and *Rhizoctonia solani* [2, 3]. These pathogens affect the root system and the basal part of the stem, which leads to a decrease in bushiness, shortening of the stem, a decrease in the number of productive shoots, and, as a consequence, significant losses in yield [4]. Root and crown rot diseases are widely distributed across major wheat‐growing regions worldwide and represent persistent constraints to yield stability under diverse agroecological conditions [5, 6]. Recent research in Kazakhstan has confirmed the widespread occurrence of these pathogens on wheat, barley, and triticale crops, highlighting their economic significance [7–11]. The current study on common root rot complements and extends this body of work by addressing another major biotic stress affecting wheat in Kazakhstan. By integrating pathogen diversity assessment, resistance genotyping, and field evaluation, this research is aimed at filling existing gaps and contributing to the development of wheat cultivars with broad‐spectrum and durable resistance.

Wheat production in warm and semiarid regions is severely constrained by a complex of fungal diseases, among which spot blotch, common root rot, and crown rot are of major importance. These diseases are particularly prevalent under conditions of elevated temperatures and fluctuating soil moisture, where pathogen pressure is often persistent and difficult to manage in the field [12]. The development and severity of spot blotch and root‐associated diseases are strongly influenced by environmental factors and agroecological conditions, resulting in pronounced regional differentiation in disease expression [13]. In response to these challenges, international breeding programs have established dedicated screening nurseries to identify wheat germplasm with stable resistance under high disease pressure across diverse environments [14]. Genetic studies have demonstrated that resistance to spot blotch and related root diseases is predominantly quantitative, governed by multiple loci with additive effects, including QTLs co‐locating with adult plant resistance genes such as *Lr34* and *Lr46* [15, 16]. *B. sorokiniana*, a key necrotrophic pathogen of wheat, is responsible for several economically important diseases, including spot blotch, black point, and common root rot, with resistance inheritance showing complex genetic control in spring wheat [17, 18]. High pathogenic and genetic variability within *B. sorokiniana* populations further complicates resistance breeding and contributes to instability of disease responses under field conditions [19]. In addition, soilborne pathogens such as *Fusarium culmorum* and *Microdochium bolleyi* have been reported as causal agents of root rot in cereals, including triticale, highlighting the expanding diversity of pathogen complexes affecting wheat production in different regions [7]. Recent phenotyping and molecular studies have also emphasized the importance of integrating genetic resistance with field‐based evaluation to improve the durability of disease control in wheat breeding programs [20, 21].

The development of root rot is closely related to the growth phases of plants. The most vulnerable are the early phases, such as tillering, when the root system is not yet sufficiently developed, and environmental conditions (temperature and soil moisture) contribute to the active spread of pathogens [22]. Similar growth stage–dependent patterns of susceptibility have been reported in other wheat‐growing regions, where early vegetative stages were identified as critical periods for root and crown rot development [23, 24]. In later stages (earing and milky ripeness), plant resistance to the disease increases, but the damage already caused can significantly affect the formation of crop yields [25]. The degree of damage by root rot varies depending on the growth phase: Maximum susceptibility is observed during the tillering phase, while during the earing and milk ripeness phases, plant resistance increases, which affects the final yield.

An important role in the spread and harmfulness of root rot is played by the background growing conditions. These include soil type, crop rotation, mineral nutrition level, and the use of agrochemicals such as fungicides [26]. Artificial contamination of soil with pathogens significantly increases the degree of plant infestation, while presowing treatment with fungicides can effectively reduce the infectious load [27]. However, long‐term use of chemical pesticides can cause resistance in pathogens and also harm plant growth due to disruption of the microbiological balance of the rhizosphere [28]. Background conditions, such as fungicide application or artificial infestation, have a significant impact on the severity of infestation and yield, especially in the early growth stages when plants are most vulnerable. In Kazakhstan, field surveys conducted across major wheat‐growing regions (Karagandy, East Kazakhstan, and Almaty) revealed that *B. sorokiniana* and *F. culmorum* were the predominant pathogens, responsible for more than 60% of crown and root rot cases and yield losses of up to 30% [8, 11, 29]. Furthermore, *R. solani* AG2‐1, *F. redolens*, and *F. pseudograminearum* have been recently reported as emerging pathogens in Kazakh agroecosystems [10, 11, 30].

Molecular surveys have revealed that Kazakhstan harbors a diverse assemblage of *Fusarium* species, including several novel taxa such as *F. campestre*, *F. kazakhstanicum*, *F. rhizicola*, and *F. steppicola*, isolated from agricultural and natural habitats [31]. The involvement of multiple *Fusarium* species in root and crown rot reflects a broader global pattern of soilborne pathogen complexes affecting cereals under different environmental conditions [32]. The identification of these new lineages underlines the evolutionary complexity and ecological adaptation of root‐associated fungi in northern Eurasia. Similarly, Alkan et al. [7] reported *F. culmorum* and *M. bolleyi* as additional causal agents of root rot on triticale, while *B. sorokiniana* remains the dominant pathogen across cereals. These findings collectively demonstrate that wheat and its relatives in Kazakhstan face a multifactorial pathogen pressure, complicating the development of effective management strategies.

An alternative approach to controlling root rot is to use resistant varieties adapted to regional growing conditions [33]. Plant resistance to pathogens is determined by a complex of genetic mechanisms, including activation of antioxidant defense, increased lignification of cell walls, and synthesis of phytoalexins [34]. Structural and biochemical defense responses, particularly cell wall reinforcement through lignification and phenylpropanoid metabolism, play a key role in limiting the spread of soilborne fungal pathogens in cereal crops [35]. Selecting varieties that combine high productivity and resistance to biotic stressors is an important element of sustainable agriculture [36].

In the Almaty Region, the combination of climatic and soil conditions characterized by elevated temperatures and humidity during the growing season—creates a favorable environment for the development of root rot. This emphasizes the importance of adapting crop management practices to regional ecological conditions. Hence, this study was conducted to assess the influence of growth stages (tillering, heading, and harvesting), cultivation backgrounds (natural, artificially infectious, and fungicidal), and disease severity on the spread of root rot and the yield formation of 16 spring wheat varieties. This research uniquely integrates phenological, environmental, and pathological parameters to evaluate the interaction between growth stages, root rot severity, and yield in the Almaty Region. The objectives were as follows: (1) to quantify disease dynamics across growth stages, (2) to assess the effects of fungicidal and infectious backgrounds, and (3) to identify resistant and high‐yielding genotypes.

## 2. Methods

The study of the influence of growth phases, background conditions, and the degree of damage on the spread of root rot and the formation of yield of spring soft wheat was carried out in 2024 in the experimental fields of the Kazakh Research Institute of Agriculture and Plant Growing, located in the village of Almalybak, Almaty Region (43.237589, 76.692629). The experiment was organized with three replications, and the area of each plot was 3 m^2^.

To assess the influence of growing conditions on the development of root rot and wheat yield, the experiment was conducted on three backgrounds: (1) plots treated with fungicides during the growing season to suppress the development of pathogens, (2) plots artificially infected with conidia of the fungus *B. sorokiniana* after sowing to create an increased infectious load, and (3) plots where plants developed without additional exposure, which corresponded to natural growth conditions. The area of the plots was 3 m^2^, and the experiment was repeated three times. Commercial varieties of spring soft wheat, Aktobe 39, alb., and Dynasty, lut., were used as standard varieties.

The prevalence and dynamics of root rot development were assessed at the phenological stages of plant development: tillering, heading, and harvesting. For this purpose, a visual analysis of 50 randomly selected plants was performed on each experimental plot. The degree of root system damage was assessed on a 4‐point scale: 0 points, *no visible symptoms of damage* (healthy plants); 1 point, *slight damage* (up to 25% of the root system is damaged); 2 points, *moderate damage* (25%–50% of the root system is damaged); and 3 points, *severe damage* (more than 50% of the root system is damaged). This assessment method made it possible to quantitatively determine the level of disease development and track its dynamics depending on the plant growth phase. The data obtained were used to analyze the effect of root rot on the productivity and condition of plants during the growing season [11].

To assess the impact of root rot on plant growth and productivity, the following measurements were taken: number of productive stems per plant, plant height (from base to tip of ear), ear length, number of ears per ear, grain weight from 50 plants, and weight of 1000 grains (calculated as the average of two samples of 500 grains each) [11, 37].

The fungicide used was Spectro Forte SC (flutriafol 160 g/L + pyraclostrobin 40 g/L; manufactured by Synthesia Chemi GmbH, Germany), applied at 0.5 L/ha as a seed treatment before sowing [38].

The prevalence of common root rot (*P*) was calculated using the formula:

P=N/n×100,

where *N* is the number of diseased plants and *n* is the total number of plants in the sample.

Root rot development (*R*) was determined using the formula:

R=A×Ka×b/K×100,

where *A* is the total number of plants in the sample, *a* is the number of plants with specific damage, *b* is the corresponding damage score, and *K* is the maximum possible damage score [11].

The harmfulness of root rot (*B*) was calculated as the percentage reduction in the yield of diseased plants compared to healthy ones using the formula:

B=a+b/a×b×100,

where *a* is the healthy plants’ yield and *b* is the diseased plants’ yield [11].

The collected data were processed using MS Excel and RStudio. The nonparametric Mann–Whitney test was used to analyze differences between groups. Data normality and variance homogeneity were tested using Shapiro–Wilk and Levene’s tests before ANOVA. Differences between means were determined using Tukey’s HSD test (*p* < 0.05). Analysis of variance (ANOVA) was used to assess the influence of factors (background and growth phase) on plant growth parameters and productivity. All data were tested for statistical significance.

## 3. Results

### 3.1. Descriptive Statistics

Descriptive statistics for common root rot infection in spring wheat are summarized in Table 1. The prevalence of the disease ranged from 0% to 100% (median = 30*%*, mean = 34.18*%*), while disease development varied between 0% and 50% (median = 7.50*%*, mean = 10.72*%*). The dataset demonstrated considerable variability across samples, confirming the heterogeneity of infection intensity among tested genotypes (Table 1).

**Table 1:** Descriptive statistics of common root rot infection indices in spring soft wheat (Kazakh Research Institute of Agriculture and Plant Growing, Almaty Region, 2024).

Minimal	1st quantile	Median	Mean	3rd quantile	Maximal
**Soft wheat, prevalence of root rot, %**					
0.00	0.00	30.00	34.18	50.00	100.00
**Soft wheat, root rot development, %**					
0.00	0.00	7.50	10.72	17.50	50.00

### 3.2. Effect of Growth Stage

**Figure 1:**
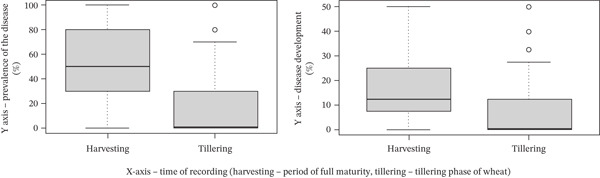
The influence of the growth phase on the spread and development of common root rot on spring soft wheat in the conditions of the Almaty Region (Kazakh Research Institute of Agriculture and Plant Growing, Almaty Region, 2024). Notes: *Y*‐axis, prevalence of the disease (%)/disease development (%); *X*‐axis, time of recording (harvesting, tillering). Statistics: *p* < 0.01 for both parameters.

Prevalence and development of common root rot in spring wheat at the tillering and harvesting stages were evaluated. Both disease parameters were significantly higher at tillering (32.65% and 8.78%, respectively) compared with harvest (11.46% and 3.61%; *p* < 0.01). Error bars represent standard errors (Figure 1, Table 2).

**Table 2:** Prevalence and development of common root rot in spring wheat at different growth stages.

Time	Spreading, %	Development, %
Harvesting stage	11.46	3.61
Tillering stage	32.65	8.78
*p* value	< 0.01	< 0.01

### 3.3. Effect of Background Conditions

**Figure 2:**
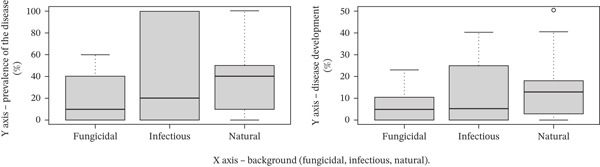
Effect of background conditions on common root rot in spring wheat (Kazakh Research Institute of Agriculture and Plant Growing, Almaty Region, 2024). Notes: *Y*‐axis, prevalence of the disease (%)/disease development (%); *X*‐axis, background (fungicidal, infectious, and natural). Statistics: *p* < 0.01 for both parameters.

Background conditions exerted a statistically significant influence on both the prevalence and development of common root rot (Table 3, Figure 2). Under fungicidal treatment, disease prevalence and development were 8.80% and 1.15%, respectively. In the infectious background, the corresponding values increased to 31.60% and 5.52%, whereas under natural conditions, they reached 26.79% and 10.22%.

**Table 3:** Effect of background conditions on the prevalence and development of common root rot in spring wheat.

Background	Prevalence, %	Development, %
Fungicidal	8.80	1.15
Infectious	31.60	5.52
Natural	26.79	10.22
*p* value	< 0.01	< 0.01

### 3.4. Grain Yield Under Different Background Conditions

**Figure 3:**
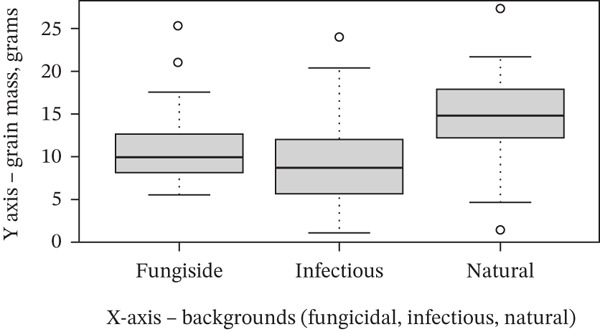
Grain weight per 10 plants of spring wheat under different backgrounds (Kazakh Research Institute of Agriculture and Plant Growing, Almaty Region, 2024). Notes: *Y*‐axis, grain mass (g); *X*‐axis, background (fungicidal, infectious, and natural). Statistics: *p* < 0.01 for both parameters.

Grain weight per 10 plants varied significantly among the tested background conditions (Table 4, Figure 3). The highest mean grain weight was recorded under natural conditions (13.71 g), followed by the fungicidal background (9.54 g), while the lowest value was observed under the infectious background (8.31 g).

**Table 4:** Grain weight per 10 plants of spring wheat under different background conditions.

Background	Weight of grain of 10 plants, g
Fungicidal	9.54
Infectious	8.31
Natural	13.71
*p* value	< 0.01

### 3.5. Biometric Traits Under Different Background Conditions

Background conditions had a significant effect on the biometric parameters of spring wheat (Table 5). Under the fungicidal background, the mean values of tillering capacity, stem length, spike length, and number of spikelets were 1.26 units, 83.43 cm, 7.39 cm, and 14.01, respectively. Under the infectious background, the corresponding values were 1.43 units, 70.18 cm, 7.59 cm, and 13.50, while under natural conditions, the parameters reached 1.81 units, 86.98 cm, 8.23 cm, and 15.21.

**Table 5:** Biometric traits of spring wheat under different background conditions of spring soft wheat in the conditions of the Almaty Region (Kazakh Research Institute of Agriculture and Plant Growing, Almaty Region, 2024).

Background	Bushiness, units	Stem length, cm	Ear length, cm	Number of spikelets, units
Fungicidal	1.26	83.43	7.39	14.01
Infectious	1.43	70.18	7.59	13.50
Natural	1.81	86.98	8.23	15.21
*p* value	< 0.01	< 0.01	< 0.01	< 0.01

### 3.6. Correlations Among Morphological Traits

Correlation analysis demonstrated significant associations among the evaluated morphological parameters (Table 6, Figure 4). A strong positive correlation was observed between spike length and the number of spikelets per spike (*r* = 0.72, *p* < 0.001). Moderate correlations were identified between tillering capacity and spike length (*r* = 0.43, *p* < 0.01), as well as between stem length and the number of spikelets (*r* = 0.46, *p* < 0.01). In addition, a weak but significant correlation was found between tillering capacity and stem length (*r* = 0.23, *p* < 0.05).

**Table 6:** Correlation between morphological characteristics of spring soft wheat: Tillering, stem length, spike length, and number of spikelets.

	Bushiness (units)	Stem length, cm	Ear length, cm	Number of spikelets (units)
Bushiness (units)		Weak connection	0.43^b^	Weak connection^a^
Stem length, cm	Weak connection		0.41^b^	0.46^b^
Ear length, cm	0.43^b^	0.41^b^		0.72^c^
Number of spikelets (units)	Weak connection^a^	0.46^b^	0.72^c^	

^a^Weak relationship.

^b^Moderate relationship.

^c^Strong relationship.

Figure 4Scatterplots showing (a) stem length vs. spikelets (*r* = 0.46) and (b) spike length vs. spikelets (*r* = 0.72). Notes: *Y*‐axis, number of spikelets (units); *X*‐axis, stem length (cm)/ear length (cm).

**Figure:**
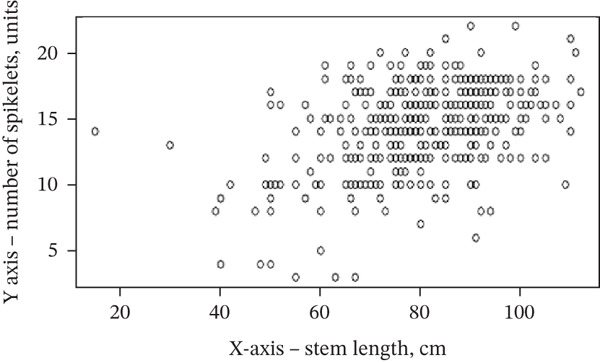
(a)

**Figure:**
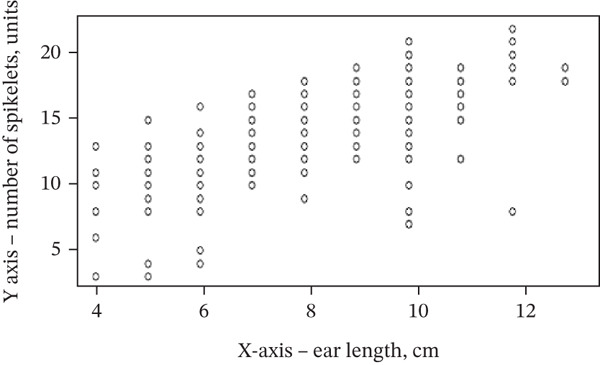
(b)

#### 3.6.1. Varietal Differences

Analysis of 16 spring wheat genotypes revealed significant variability across all measured biometric traits (Table 7). The highest mean values for stem length, spike length, and number of spikelets were recorded in No. 445/Chelaba 80 (lut.) (87.45 cm, 9.15 cm, and 16.40, respectively). Ekada 113 (alb.) also exhibited superior morphological characteristics (81.60 cm, 8.10 cm, and 15.57), followed by No. 459/Saratovskaya 35 (velut.) (85.87 cm, 7.87 cm, and 15.23). The standard variety Aktobe 39 (alb.) showed stable and balanced performance (83.90 cm, 7.83 cm, and 14.77). In contrast, Liniya R‐1413m displayed the lowest spike length (6.30 cm) and spikelet number (12.80), indicating relatively weaker morphological development compared with the other genotypes.

**Table 7:** Biometric characteristics of 16 spring wheat lines (Kazakh Research Institute of Agriculture and Plant Growing, Almaty Region, 2024).

Line/variety	Bushiness, units	Stem length, cm	Ear length, cm	Number of spikelets, units.
No. 401/k‐52304 WW14753, lut.	1.77	68.54	7.23	13.73
No. 410/k‐57729 Tselinnaya yubil., lut.	1.87	79.17	7.70	14.27
No. 436/Liniya 1616 ae 14, lut.	1.43	82.37	7.90	14.70
No. 445/Chelaba 80, lut.	1.55	87.45	9.15	16.40
No. 448/Orenburgskaya 23, lut.	1.68	78.23	8.85	15.10
No. 449/Orenburgskaya Ubileinaya	1.43	73.80	7.47	13.23
No. 459/k‐43285 Saratovskaya 35, velut.	1.30	85.87	7.87	15.23
Aktobe 39, alb. standard	1.60	83.90	7.83	14.77
Dynastiya, lut. standard	1.27	75.23	7.03	14.13
Liniya 1415m	1.38	83.42	8.19	14.88
Liniya 201m	1.67	83.20	7.60	11.90
Liniya 205m	1.20	84.80	7.90	13.77
Liniya R‐1413m	1.17	80.73	6.30	12.80
Stepnaya 2, lut.	1.63	83.07	7.50	14.67
Stepnaya 50, alb.	1.60	76.47	7.37	13.47
Ekada 113, alb.	1.53	81.60	8.10	15.57
*p* value	<0.01	<0.01	<0.01	<0.01

### 3.7. Effect of Damage Degree

Biometric traits of spring wheat varied significantly with the degree of infection (Table 8). Plants exhibiting first‐degree infection had the lowest values for tillering capacity (1.47 units), stem length (73.95 cm), spike length (7.49 cm), and number of spikelets (13.71). At the second degree of infection, these parameters slightly increased to 2.33 units, 75.67 cm, 7.67 cm, and 14.67, respectively. The highest mean values were recorded in healthy plants, with tillering capacity of 1.53 units, stem length of 86.71 cm, spike length of 7.99 cm, and 14.79 spikelets per spike.

**Table 8:** Biometric indicators of spring wheat depending on damage degree (*p* < 0.01).

Indicator	Tillering (units)	Stem length (cm)	Spike length (cm)	Spikelets per spike
1st degree	1.47	73.95	7.49	13.71
2nd degree	2.33	75.67	7.67	14.67
Healthy	1.53	86.71	7.99	14.79
*p* value	< 0.01	< 0.01	< 0.01	< 0.01

Table 1 presents the results of descriptive statistics of the indices of spring soft wheat infection with common root rot obtained in the Almaty Region (Kazakh Research Institute of Agriculture and Plant Growing, 2024). Data analysis shows significant variability in the prevalence and development of the disease. The prevalence of root rot varied from 0% to 100%, with the median value being 30% and the mean value (mean) being 34.18%. This indicates that, on average, about a third of the plants in the studied sample were susceptible to the disease. The wide range of values (from minimum to maximum) can be due to both the genetic characteristics of the studied varieties and the influence of external factors, including agroclimatic conditions, soil type, and the level of agricultural technology.

Root rot development also showed significant variability, ranging from 0% to 50%, with a median of 7.50% and an average of 10.72%. This suggests that the disease was mild to moderate in most cases but could reach high intensity (up to 50%) in some cases. The findings highlight the need for further study of factors contributing to root rot development, including the analysis of varietal resistance, the impact of agronomic practices, and the effectiveness of fungicide application. The results highlight the need to develop comprehensive protection measures to reduce the negative impact of the disease on spring soft wheat yields in the region.

## 4. Discussion

The results of the study demonstrate significant variability in the prevalence (0%–100%) and development (0%–50%) of common root rot in spring soft wheat in the Almaty Region. These findings are consistent with those of Bozoglu et al. [8], who reported that disease intensity in Kazakhstan varies with agroclimatic factors and the genetic makeup of cultivars. In our study, the average prevalence was 34.18%, indicating high pathogen activity in this environment. Similar observations were made by Ozer et al. [9] and Alkan et al. [7], who emphasized that the early growth phases, especially tillering, are the most vulnerable to infection. This corresponds with our results, where prevalence reached 32.65% and disease development 8.78% at the tillering stage. Similar patterns were reported in China [3] and Chile [25], where early‐stage infection by *B. sorokiniana* resulted in 20%–30% yield losses.

The effectiveness of fungicide application in reducing disease prevalence (8.80%) and development (1.15%) is consistent with previous findings [11], which demonstrated the value of chemical protection against *B. sorokiniana* and *Fusarium* spp. in southeastern Kazakhstan.

However, similar to our data, they noted that fungicide use can induce plant stress and suppress growth, leading to reduced productivity. This may explain why grain weight under the fungicide background (9.54 g) was lower than under the natural background (13.71 g). Bozoglu et al. [8] also recommended combining fungicides with agronomic and biological approaches to mitigate stress effects and maintain a balanced rhizosphere.

Higher yield under natural conditions (13.71 g) likely reflects favorable soil and environmental factors and the absence of chemical stress when infection pressure is moderate. This pattern corresponds with the findings of Bozoglu et al. [8], who reported that host defenses can maintain productivity under low pathogen load. However, the yield significantly decreased (8.31 g) under the infectious background, emphasizing the necessity of using resistant cultivars and implementing integrated disease management (IDM). The importance of such approaches has been highlighted by Dutbayev et al. [29] and Kharipzhanova et al. [11], who showed that crop rotation, soil treatment, and the use of biopreparations effectively reduce infection pressure and improve sustainability.

The strong correlation between spike length and spikelet number (*r* = 0.72) observed in this study confirms that these traits play a key role in yield formation under biotic stress [8, 11]. Moderate correlations between tillering, stem length, and spikelet number are consistent with the integrated breeding approach proposed by Akhmetova et al. [31], emphasizing the importance of selecting genotypes with optimized architecture and resistance. Recent surveys [7, 9, 10, 30] revealed that the pathogen complex affecting wheat in Kazakhstan includes *B. sorokiniana*, *F. culmorum*, *F. pseudograminearum*, *F. redolens*, *M. bolleyi*, and *R. solani* AG2‐1, which complicates disease management and increases the risk of yield losses.

In this context, the observed relationships between morphological traits and disease response are consistent with earlier breeding‐oriented studies conducted under contrasting agroecological conditions in Kazakhstan. Previous research has shown that wheat genotypes combining favorable morphological and yield‐related traits with genetic resistance to major fungal diseases tend to maintain higher productivity under biotic stress. These studies highlighted the importance of integrating phenotypic selection with field‐based and molecular evaluations to identify stable and adaptable genotypes. The agreement between our findings and these reports supports the relevance of traits such as spike length and tillering capacity as indirect indicators of yield resilience under disease pressure [39–41].

These results are further supported by recent studies focusing on the genetic and phenotypic basis of disease resistance in spring wheat. Previous research has shown that genotypes combining favorable morphological traits with resistance to fungal pathogens exhibit enhanced yield stability under biotic stress conditions. These studies emphasized the value of integrating phenotypic assessments with molecular tools to identify wheat lines capable of maintaining productivity across contrasting environments, which is consistent with the trait associations observed in the present study [21, 42].

The superior performance of varieties No. 445/Chelaba 80 and Ekada 113 in stem and spike traits highlights their potential for regional adaptation. This observation is consistent with previous findings [8, 11], which demonstrated that resistant cultivars can substantially reduce yield losses caused by soilborne pathogens. Conversely, the weak performance of the variety Liniya R‐1413m highlights the need for continued investigation into the physiological and genetic mechanisms underlying resistance across contrasting agroecological backgrounds [8, 31].

The reduction in yield under fungicide treatment may be associated with alterations in the rhizosphere microbiota and suppression of beneficial fungi [27, 43].

Overall, the results confirm the importance of IDM that combines resistant varieties, rational fungicide use, and adaptive agronomic practices. Given the expanding diversity of soilborne pathogens in Kazakhstan—including *B. sorokiniana*, *F. culmorum*, *F. redolens*, *F. pseudograminearum*, *M. bolleyi*, and *R. solani* AG2‐1 [7–9, 11, 30, 31]—future research should integrate molecular diagnostics, genotype‐by‐environment analyses, and region‐specific IDM strategies to enhance the resilience of wheat production systems in the region.

## 5. Conclusions

The study revealed substantial variability in the prevalence and development of common root rot in spring soft wheat across the Almaty Region. The highest infection levels were recorded during the tillering stage, confirming its critical importance for disease prevention. Fungicide application effectively suppressed infection but was accompanied by reduced grain weight, likely due to chemical stress. Natural background conditions ensured better plant growth and yield parameters, while the infectious background caused the most severe losses. Strong correlations between spike length and spikelet number underline their role as key productivity traits under disease pressure. The results confirm the need for early‐stage monitoring and integrated protection strategies to mitigate root rot damage. Resistant varieties such as Chelaba 80 and Ekada 113 demonstrated superior adaptation and can serve as valuable material for breeding programs aimed at enhancing wheat resilience in Kazakhstan.

## Funding

This study was funded by the Ministry of Education and Science of the Republic of Kazakhstan, 10.13039/501100004561, no. AP26195327, Genome‐Wide Association Mapping of Resistance to Common Root Rot Caused by *Bipolaris sorokiniana* in Wheat Germplasm From Kazakhstan.

## Conflicts of Interest

The authors declare no conflicts of interest.

## Data Availability

The data that support the findings of this study are available from the corresponding author upon reasonable request.
